# Characterization of Abelmoschus esculentus plant waste fiber for sustainable composite and biomedical applications

**DOI:** 10.1038/s41598-026-39438-y

**Published:** 2026-02-13

**Authors:** Thandavamoorthy Raja, Yuvarajan Devarajan, Aravindan Munusamy Kalidhas, G. M. Sandeep, Mukul Saxena, Sasanka Choudhury, D. Dhorajiya Amitkumar, Kulmani Mehar

**Affiliations:** 1 Materials Science Lab, Department of Prosthodontics, Saveetha Dental College and Hospitals, SIMATS, Chennai, India; 2 Department of Mechanical Engineering, Saveetha School of Engineering, SIMATS, Chennai, Tamil Nadu India; 3 Department of Mechanical Engineering, Faculty of Engineering and Technology, JAIN (Deemed-to-be University), Ramanagara District, Karnataka, 562112 India; 4 Department of Mechanical Engineering, Presidency University, Bengaluru, Karnataka India; 5 Department of Mechanical Engineering, Noida Institute of Engineering & Technology, Greater Noida, Uttar Pradesh India; 6 Department of Mechanical Engineering, Siksha ’O’ Anusandhan (Deemed to be University), Bhubaneswar, Odisha India; 7 Department of Mechatronics Engineering, Faculty of Engineering and Technology, Parul Institute of Technology, Parul University, Vadodara, Gujarat India; 8 Department of Mechanical and Industrial Engineering, Manipal Institute of Technology, Manipal Academy of Higher Education, Manipal, India

**Keywords:** Sustainable development, Fibers, Antimicrobial activity, Biomedical applications, Biotechnology, Chemistry, Materials science, Microbiology

## Abstract

The valorization of agricultural waste as a source of sustainable materials is gaining momentum in composite development. This study focuses on fibers extracted from Abelmoschus esculentus (okra) plant waste stems through water retting, mechanical scraping, and alkali treatment, followed by comprehensive characterization. X-ray diffraction analysis revealed a semi-crystalline structure with a crystallinity index of 30.3%, while Fourier-transform infrared spectroscopy confirmed the presence of cellulose, hemicellulose, and lignin functional groups. Tensile testing demonstrated a tensile strength of 13.79 MPa and an elongation at break of 0.36 indicating good mechanical performance for biodegradable composites. Scanning electron microscopy showed rough and fibrillated fiber surfaces, suggesting enhanced interfacial adhesion potential. Antibacterial testing against Salmonella typhimurium exhibited a notable inhibition zone of 23 mm at a 50 µg concentration, closely comparable to the standard antibiotic streptomycin. Confocal laser scanning microscopy further revealed significant disruption of biofilm formation, with increased bacterial cell death after fiber extract treatment. These findings collectively highlight that Abelmoschus esculentus fibers possess an advantageous combination of mechanical strength, chemical functionality, and antimicrobial activity, making them efficient material for eco-friendly composite applications in environmental and biomedical sectors.

## Introduction

Sustainable development in industries is essential to balance economic growth with environmental protection and social responsibility^1^. As industrial activities significantly contribute to resource depletion, pollution, and environmental degradation, adopting sustainable practices helps mitigate these negative impacts, ensuring the availability of resources for future generations^2^. Furthermore, sustainable industrial practices enhance operational efficiency, reduce costs through energy conservation and waste reduction, and improve corporate reputation among stakeholders and consumers^3^. By integrating sustainability into their core strategies, industries not only comply with regulatory requirements but also foster innovation, competitiveness, and resilience against environmental and economic uncertainties^4^. Ultimately, sustainable development in industries promotes inclusive growth, long-term profitability, and a healthier environment, benefiting society as a whole. Natural fibers have become integral to sustainable development in polymer composite industries, notably in manufacturing sectors such as children’s toys, bicycles, and automotive components^5^. Derived from renewable resources like jute, flax, hemp, sisal, coir, bamboo, and kenaf, these fibers offer significant environmental benefits, including biodegradability, reduced carbon footprint, and lower energy consumption during production. Additionally, natural fiber composites provide advantageous mechanical properties, such as high strength-to-weight ratios, effective noise and vibration dampening, enhanced recyclability, and improved safety^6^. Utilizing these sustainable materials supports eco-friendly product development, aligns industrial processes with global sustainability objectives, and meets increasing consumer demand for environmentally responsible and safe products. Ultimately, incorporating natural fibers into polymer composites promotes a greener manufacturing paradigm, fostering long-term ecological balance and sustainable economic growth^7^. Natural fibers extracted from plant waste by-products represent an innovative and sustainable approach to material sourcing in composite industries. Agricultural and industrial residues, such as rice straw, sugarcane bagasse, banana pseudo-stems, pineapple leaves, coconut husks, and corn stalks, are abundant and often discarded or burned, leading to environmental issues^8^. Extracting fibers from these by-products not only adds economic value to otherwise discarded materials but also mitigates waste generation, reduces pollution, and enhances resource efficiency. Techniques for extraction typically include retting, mechanical decortication, chemical processing, and enzymatic treatments, each designed to separate fibers effectively while minimizing environmental impacts^9^. These recovered natural fibers exhibit desirable properties such as adequate mechanical strength, biodegradability, and lightweight characteristics that make them suitable for reinforcing polymer composites. Thus, utilizing plant-derived waste fibers in composite manufacturing promotes circular economy principles, encourages responsible waste management, and significantly contributes to the sustainable growth of industries^10^. Abelmoschus esculentus fiber, commonly known as okra fiber, exhibits favourable properties that make it suitable for composite applications. Typically, these fibers possess a low density, ranging approximately between 1.15 and 1.45 g/cm³, facilitating lightweight composite structures. Their diameter generally varies from 50 to 250 micrometres, providing sufficient surface area for effective interfacial bonding within polymer matrices^11^. Moreover, the fibers demonstrate good thermal stability and biodegradability, positioning them as environmentally sustainable alternatives to synthetic fibers^12^. These collective properties make Abelmoschus esculentus fibers increasingly attractive for developing eco-friendly composite materials in various industrial sectors. Investigating the functional and antimicrobial properties of natural fibers is essential to enhance their applicability, safety, and overall value in industrial and consumer products^13^.

Natural fibers, despite their eco-friendly and biodegradable nature, can be susceptible to microbial attack, leading to deterioration in mechanical strength, durability, and hygienic properties. By assessing their inherent antimicrobial capabilities or exploring modifications to improve these attributes, researchers can develop safer, more durable, and hygienically superior composite materials^14^. Additionally, identifying and optimizing functional properties such as moisture absorption, UV resistance, thermal stability, and flame retardancy broadens the potential application areas of these fibers, particularly in sectors such as healthcare, packaging, textiles, automotive interiors, and children’s products^15^. Ultimately, examining these characteristics contributes to increased sustainability, consumer safety, product longevity, and market competitiveness of natural fiber-based composites^16^. Integrating natural fibers into the United Nations’ Sustainable Development Goals (SDGs) represents a meaningful strategy for achieving environmental sustainability, economic growth, and social well-being^17^. Utilizing renewable natural fibers aligns directly with SDGs such as Responsible Consumption and Production (SDG 12) by reducing dependency on non-renewable resources and minimizing waste through the valorisation of agricultural by-products^18^. Their biodegradability and reduced carbon footprint significantly contribute to Climate Action (SDG 13), while fostering innovation and industrial sustainability supports Industry, Innovation, and Infrastructure (SDG 9). Moreover, promoting natural fibers from local agricultural communities can enhance livelihoods, reduce poverty (SDG 1), and support Decent Work and Economic Growth (SDG 8), particularly in rural areas^19^. Encouraging sustainable cultivation and extraction practices of natural fibers further promotes Life on Land (SDG 15) by protecting ecosystems and biodiversity. Thus, strategically merging natural fibers with SDG objectives can accelerate the global transition toward a sustainable, equitable, and environmentally responsible future^20,21^.

This study aims to investigate the use of natural fibers extracted from the stem waste of *Abelmoschus esculentus* plant as reinforcement materials in polymer composites. The research specifically focuses on characterizing these fibers for their antibacterial properties and biocompatibility through advanced analytical techniques, including X-ray diffraction (XRD), Fourier-transform infrared spectroscopy (FTIR), and scanning electron microscopy (SEM). In addition, the study explores the feasibility of integrating these fibers into the fabrication of automotive components, with the broader objective of reducing the need for extensive material sanitation processes while enhancing the performance, durability, and environmental sustainability of the composites.

## Materials and experimental process

### Fiber extraction process

Abelmoschus esculentus (okra) stem waste was collected from Sri Venkatesa Agro Farm, a privately owned agricultural field in Periyagaram Village, Tiruvannamalai District, Tamil Nadu, India. The stems were obtained as post-harvest agricultural residues with the landowner’s consent and were neither purchased nor sourced from protected or regulated areas. After collection, the stems were washed thoroughly with tap water to remove adhering soil and impurities and cut into uniform lengths of approximately 30–40 cm to ensure consistent processing. Fiber extraction was carried out through a sequential process involving water retting, mechanical separation, alkali treatment, neutralization, and drying. Water retting was performed by immersing the cleaned okra stems in freshwater at ambient temperature (28 ± 2 °C). A material-to-water ratio of approximately 1:20 (w/v) was maintained to ensure uniform microbial activity. Retting was continued for 12 days, during which naturally occurring anaerobic microorganisms decomposed pectins, hemicellulose, and other cementing substances binding the fibers to the woody core. The retting progress was monitored daily to avoid over-retting, which can lead to cellulose degradation and loss of fiber strength.

**Fig. 1:**
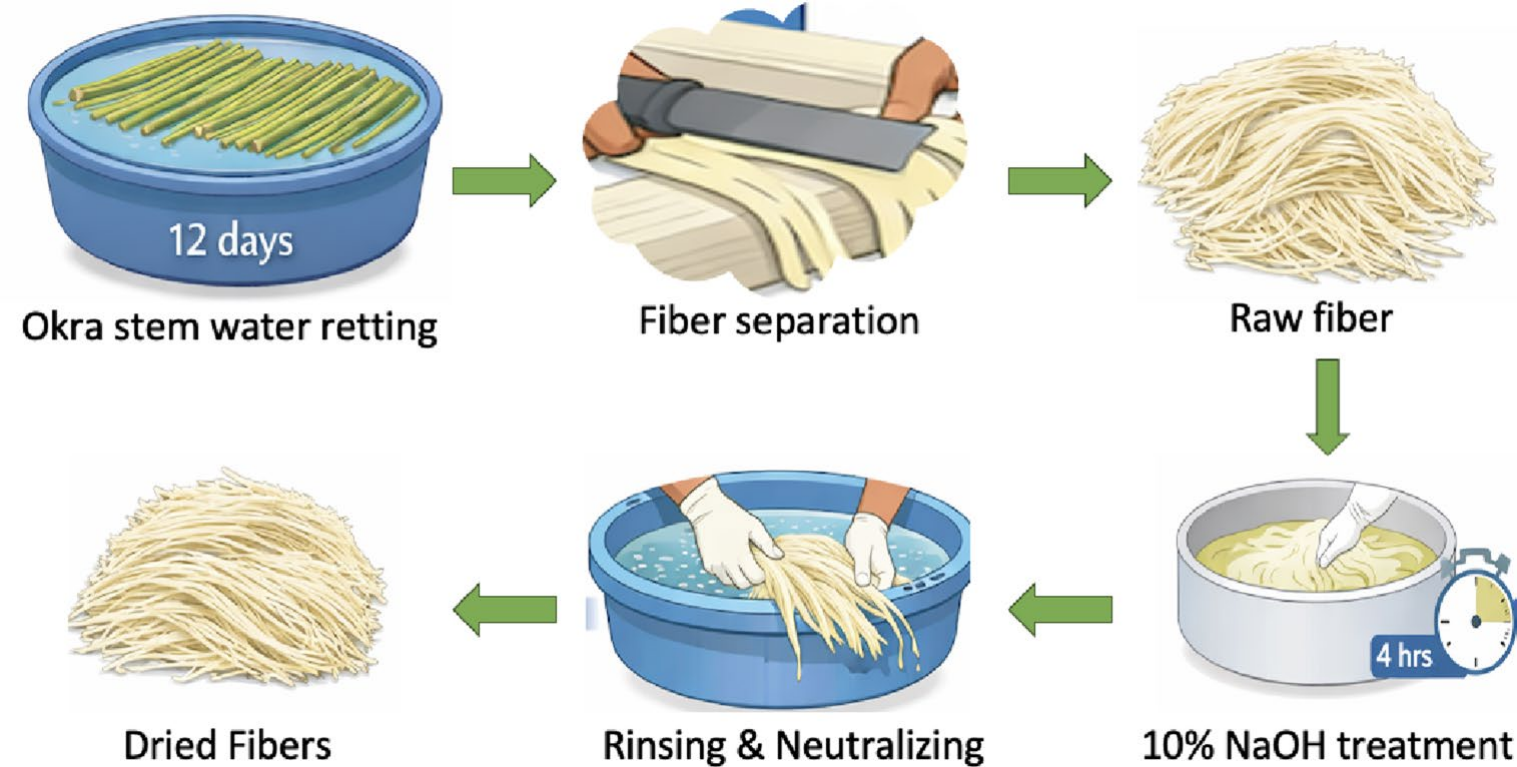
Extraction process of *Abelmoschus esculentus* fibers.

After retting, the softened stems were removed and rinsed with water, followed by mechanical scraping using a blunt steel blade to separate the fibrous bundles from the woody core. This step effectively removed residual bark and non-fibrous tissues while preserving fiber continuity and minimizing structural damage. The extracted fibers were washed repeatedly with distilled water to eliminate loosely bound impurities. Alkali treatment was carried out using a 10 wt% sodium hydroxide solution, corresponding to 2.5 M NaOH. The fibers were immersed in the alkali solution at room temperature (30 ± 2 °C) for 4 h with a fiber-to-solution ratio of 1:15 (w/v). This treatment removed residual lignin, hemicellulose, pectin, waxes, and oils, leading to cellulose enrichment and improved surface reactivity. The alkali-modified fibers exhibited increased surface roughness, which is beneficial for interfacial adhesion in polymer composites. Following alkali treatment, the fibers were thoroughly washed with distilled water until neutral pH (around 7) was achieved, as verified using a digital pH meter. This neutralization step was necessary to remove residual sodium hydroxide and prevent fiber degradation. The fibers were then dried under natural shade conditions at 30–35 °C for 48 h, followed by oven drying at 60 °C for 6 h to remove residual moisture without inducing thermal damage. The overall fiber yield obtained from okra stem waste was 28–32% by dry weight, depending on stem maturity and retting efficiency. This extraction route enables the recovery of high-quality Abelmoschus esculentus fibers with improved chemical purity, structural integrity, and surface functionality. Figure 1 shows the extraction process of *Abelmoschus esculentus* fibers.

### Testing process of *Abelmoschus esculentus* fibers

A comprehensive experimental evaluation was conducted to characterize the structural, mechanical, chemical, and biological properties of Abelmoschus esculentus fibers, following internationally accepted ASTM and CLSI standards. To assess the crystalline structure, X-ray diffraction (XRD) analysis was performed using a Bruker D8 Advance diffractometer operating at 40 kV and 40 mA with Cu-Kα radiation (λ = 1.5406 Å). The samples were scanned across a 2θ range of 5° to 80° at a scanning rate of 2° per minute. The crystallinity index was calculated using the Segal empirical method to quantify the relative amount of crystalline versus amorphous regions within the fibers. For chemical group identification, Fourier transform infrared spectroscopy (FTIR) was conducted using a PerkinElmer Spectrum Two FTIR spectrometer within the range of 4000–450 cm⁻¹, employing the potassium bromide (KBr) pellet technique. FTIR analysis confirmed the removal of non-cellulosic components such as hemicellulose, lignin, and waxes following alkali treatment with 10% sodium hydroxide (NaOH). Mechanical properties of the fibers were evaluated according to ASTM D3822-14 standards for single fiber tensile testing. Fiber specimens with an approximate gauge length of 20 mm and diameters ranging between 50 and 150 μm were mounted on paper frames to ensure alignment^22^. Tensile testing was performed on a Tinius Olsen H10KT universal testing machine, equipped with a 10 N precision load cell, at a crosshead speed of 5 mm/min. A minimum of 20 fibers (*n* = 20) was tested to ensure statistical significance. Key mechanical parameters, including tensile strength, Young’s modulus, and elongation at break, were derived from the stress-strain curves. Scanning electron microscopy (SEM) was employed to study the surface morphology and microstructural features of the extracted fibers. Imaging was performed using a ZEISS EVO 18 SEM, following gold sputter-coating with a Quorum Q150R S sputter coater to prevent charging under the electron beam. SEM analysis revealed surface texture, fibrillation, and any surface defects resulting from retting, mechanical, or chemical treatments^23^. The antibacterial activity of the fibers was investigated against Salmonella typhimurium using the agar well diffusion method, following Clinical and Laboratory Standards Institute (CLSI) protocols. Mueller-Hinton agar plates were inoculated with a bacterial suspension adjusted to a 0.5 McFarland standard (approximately 1.5 × 10⁸ CFU/mL). Wells of 6 mm diameter were bored into the agar, and 100 µL of fiber extract, prepared by soaking fibers in sterile distilled water for 24 h, was added to each well. After 24 h of incubation at 37 °C, zones of inhibition were measured to assess antibacterial efficacy. All antibacterial experiments were performed in triplicate (*n* = 3), and the inhibition zone values are reported as mean values to ensure reproducibility and reliability of the results. Additionally, biofilm inhibition analysis was performed using confocal laser scanning microscopy (CLSM) to visualize and quantify the effect of fiber extracts on bacterial biofilm formation. Biofilms were developed on sterile glass coverslips treated with fiber extracts, stained with the LIVE/DEAD BacLight bacterial viability kit (Molecular Probes, USA), and visualized under a Leica TCS SP8 CLSM. Laser excitation wavelengths of 488 nm and 561 nm were used to distinguish live and dead bacterial populations. Images were processed and analyzed using LAS X software to determine biofilm thickness, density, and viability^24^. Throughout all mechanical and microbiological tests, environmental conditions were strictly controlled. Mechanical tests were performed at 23 ± 2 °C and 50 ± 5% relative humidity, while biological assays were conducted under aseptic conditions inside a Class II biosafety cabinet.

## Results and discussion

### XRD analysis of *Abelmoschus esculentus* fiber

**Fig. 2:**
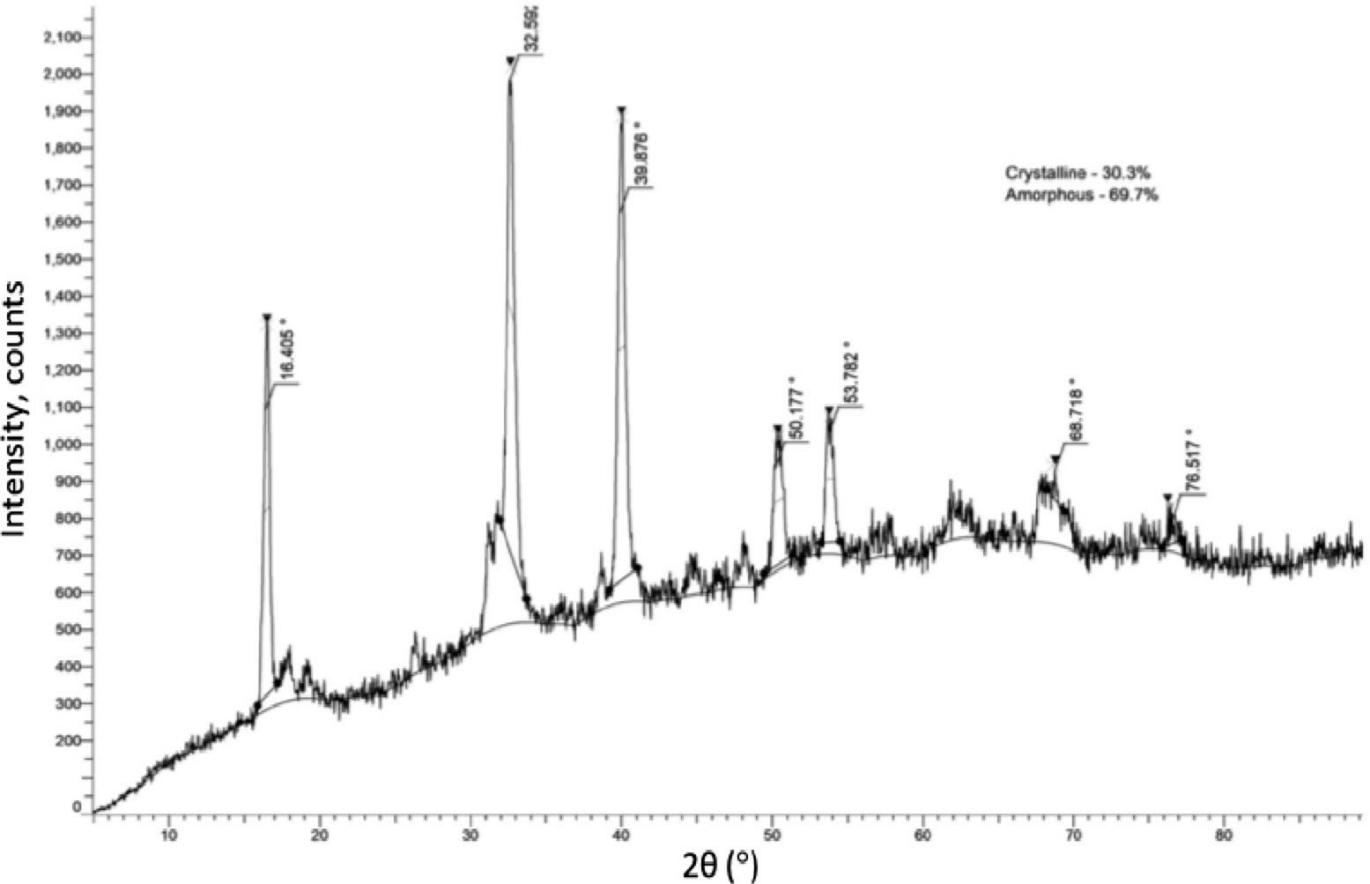
XRD analysis of *Abelmoschus esculentus* fibers.

The crystalline structure of *Abelmoschus esculentus* fibers was investigated using X-ray diffraction (XRD) analysis with Cu-Kα radiation (λ = 1.5406 Å), providing critical insights into the molecular organization of the fibers. The XRD diffractogram displays several distinct peaks alongside a broad background, type of natural lignocellulosic fibers that exhibit a semi-crystalline character. Major diffraction peaks were observed at 2θ angles of 16°, 22°, 39°, 50°, 53°, 68°, and 76°. The sharp and intense reflection at 22° corresponds to the (002) crystalline plane of cellulose I, the dominant crystalline polymorph present in plant fibers. The diffraction peaks at lower angles (around 16°) are associated with the (101) plane, indicating the microfibrillar structure of cellulose chains, while the minor peaks at higher angles represent ordered but less dominant lattice orientations and possibly traces of mineral or lignin-based residues. The crystallinity index (CI) of the okra fibers was determined using the Segal method by evaluating the intensity difference between the crystalline and amorphous regions in the X-ray diffraction pattern. Distinct diffraction peaks were observed at 2θ ≈ 16.4° and 22.6°, corresponding to the (1–10)/(110) and (200) crystallographic planes of cellulose I, respectively, with a weaker reflection near 2θ ≈ 34.9° attributed to the (004) plane. Using the intensity of the (200) peak and the minimum intensity of the amorphous background, the CI was calculated to be 30.3%, while the remaining 69.7% represents the amorphous fraction. The presence of well-defined cellulose I reflections together with a broad amorphous halo confirms the semi-crystalline nature of the okra fibers. This moderate crystallinity, arising from biological retting and mild alkali treatment, provides a balanced combination of stiffness and flexibility and is advantageous for achieving effective interfacial bonding in composite and bio-based material applications. Figure 2 shows the XRD analysis of *Abelmoschus esculentus* fibers.

The interplay between crystalline and amorphous regions plays a significant role in defining the functional properties of Abelmoschus esculentus fibers. The crystalline regions contribute substantially to the mechanical strength, thermal stability, and chemical resistance of the fibers. The tightly packed cellulose chains within these domains form hydrogen-bonded microfibrils that are difficult to hydrolyse and offer structural rigidity, which is advantageous when the fibers are used as reinforcements in polymer composites. Conversely, the high proportion of amorphous content imparts greater flexibility, improved moisture absorption, and enhanced accessibility for chemical modifications. Amorphous domains, being less ordered, present more free hydroxyl groups exposed on the fiber surface, facilitating interactions with matrices, functional molecules, or biological agents. These regions also allow fibers to swell in the presence of water, which can be beneficial in applications such as biomedical scaffolds where hydration and biocompatibility are important. However, a high amorphous content also renders fibers more susceptible to enzymatic degradation and environmental wear over time, which needs to be considered in material design depending on the intended application. The broad halo observed between 15° and 30° 2θ in the diffractogram highlights the amorphous contribution, arising from randomly oriented cellulose chains and the presence of hemicellulose and lignin that interfere with perfect crystalline packing. The minor peaks at higher 2θ values could be attributed to slight crystalline order within non-cellulosic components or residual inorganic salts remaining after extraction^25^. Therefore, the XRD results suggest that Abelmoschus esculentus fibers possess a semi-crystalline structure dominated by a significant amorphous matrix, providing a balanced combination of mechanical strength and chemical flexibility. This unique structural organization enhances their suitability for eco-friendly composite materials, packaging solutions, bio-based textiles, and biomedical devices where both durability and biodegradability are required. Importantly, the moderate crystallinity also suggests that further post-treatment processes, such as mild chemical modifications or physical treatments, could be optimized to tailor the fiber properties for specific high-performance applications.

### FTIR spectroscopic analysis of *Abelmoschus esculentus* fiber

**Fig. 3:**
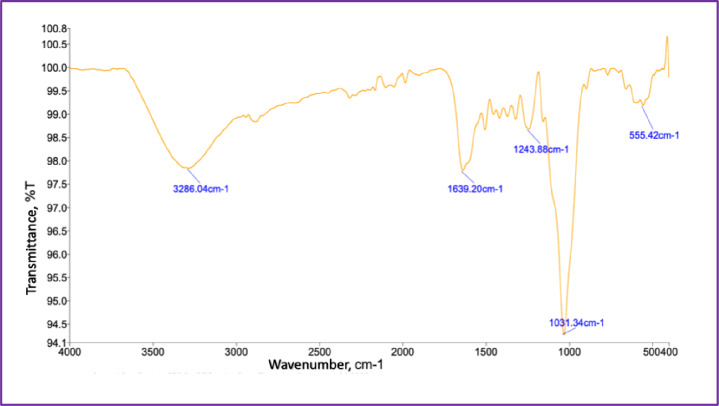
FTIR spectrum of *Abelmoschus esculentus* fibers.

The chemical composition and molecular structure of *Abelmoschus esculentus* fibers were examined using Fourier-transform infrared spectroscopy over the wavenumber range of 4000–400 cm⁻¹, as presented in Fig. 3. The spectrum, plotted in the conventional format with decreasing wavenumber from left to right, exhibits characteristic transmittance bands typical of lignocellulosic plant fibers, confirming that the material is predominantly cellulose-based with contributions from hemicellulose and lignin. A broad and intense transmittance band cantered at 3286 cm⁻¹ is attributed to O–H stretching vibrations of hydroxyl groups abundantly present in cellulose and hemicellulose. The significant breadth of this band reflects extensive intra- and intermolecular hydrogen bonding, indicating the coexistence of crystalline cellulose domains and amorphous regions within the fiber structure, which collectively influence moisture affinity, thermal stability, and interfacial behavior in composite systems. The transmittance band observed at 1639 cm⁻¹ is associated with H–O–H bending vibrations of absorbed water, with possible overlap from aromatic C = C stretching of lignin, highlighting the hydrophilic nature of the fiber matrix and the retention of phenolic structural units^26^. In the fingerprint region, a distinct band at 1243 cm⁻¹ corresponds to C–O stretching vibrations of acetyl and aryl–ether linkages, originating from hemicellulose and lignin components, suggesting that non-cellulosic constituents remain integrated within the cellulose framework and contribute to fiber flexibility and chemical heterogeneity. The most intense transmittance peak at 1031 cm⁻¹ is characteristic of C–O and C–O–C stretching vibrations associated with β-1,4-glycosidic linkages in cellulose, confirming the preservation of the cellulose backbone and its dominant role in governing the structural integrity of the fibers^27^. The low-wavenumber band observed at 555 cm⁻¹ is attributed to skeletal vibrations and out-of-plane bending modes of the cellulose backbone and aromatic structures, further supporting the presence of an organized lignocellulosic network. Collectively, the FTIR spectral features demonstrate that *Abelmoschus esculentus* fibers possess a well-retained cellulose-rich architecture interspersed with hemicellulose and lignin, resulting in a balanced combination of ordered crystalline regions that impart mechanical strength and amorphous domains that enhance flexibility, moisture interaction, and chemical accessibility. The abundance of hydroxyl and ether functional groups provides active sites for hydrogen bonding and interfacial interactions with polymer matrices, while residual lignin contributes to structural rigidity and partial hydrophobicity, thereby confirming the suitability of these fibers for advanced applications in sustainable composites, biodegradable packaging, and bio-functional textile materials.

### Tensile strength of *Abelmoschus esculentus* fiber

**Fig. 4:**
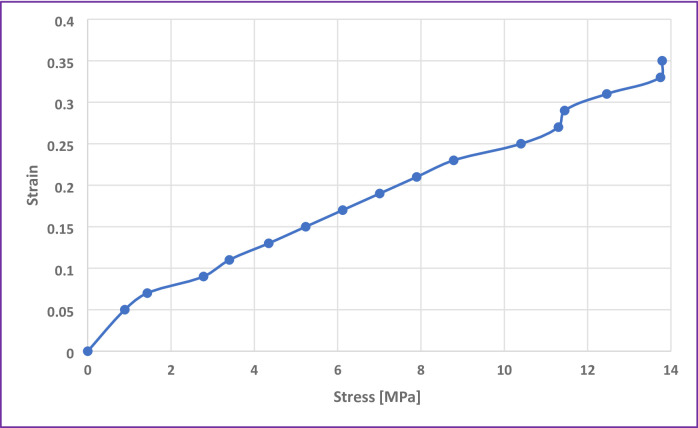
The stress vs. strain curve of *Abelmoschus esculentus* fibers.

The tensile properties of *Abelmoschus esculentus* fiber were systematically evaluated to assess the single fiber mechanical performance, particularly focusing on their stress–strain behavior under uniaxial loading. The stress-strain curve exhibited a pattern for natural fibers, characterized by an initial linear elastic region followed by a gradual yielding phase leading to failure. The fiber demonstrated a tensile strength of 13.79 ± 1.5 MPa, highlighting their capacity to endure substantial mechanical loads before rupture. The initial portion of the curve showed a nearly linear relationship between stress and strain, indicating elastic deformation governed by the stretching of molecular bonds, primarily within the crystalline regions of cellulose. From the slope of this linear region, the elastic modulus can be estimated, reflecting the stiffness of the fibers^28^. Beyond the elastic limit, a gradual non-linear increase in strain occurred with increasing stress, suggesting microstructural rearrangements within the fiber matrix, such as the slippage of cellulose microfibrils and disruption of amorphous domains. The fibers exhibited a maximum strain (elongation at break) of 0.36, indicating brittle nature of natural fibers. This combination of moderate tensile strength and brittle suggests that the Abelmoschus esculentus fibers possess a well-balanced microstructure, consisting of both crystalline cellulose domains providing strength and amorphous regions, such as hemicellulose and residual lignin, contributing to flexibility and energy dissipation under tensile load. Figure 4 shows the stress vs. strain curve of *Abelmoschus esculentus* fibers.

The observed tensile strength of 13.79 MPa, while lower compared to highly processed synthetic fibers, falls within the acceptable range for many biodegradable and natural fiber applications. It demonstrates the fiber’s suitability for use in low to moderate load-bearing applications such as eco-friendly composites, bioplastics reinforcement, nonwoven fabrics, and sustainable packaging materials. Moreover, considering that the fibers underwent only basic mechanical and chemical treatments (retting, mechanical scraping, and alkali treatment), the tensile properties could be further enhanced by surface treatments, fiber alignment, or composite processing techniques. Mechanistically, the moderate tensile strength can be explained by the interplay of fiber microfibrillar angle, cellulose crystallinity (30.3% as observed from XRD analysis), and the presence of amorphous matrices. A higher microfibril angle correlates with greater extensibility but lower strength, while moderate crystallinity contributes to maintaining a balance between rigidity and slight ductility. The amorphous components, while reducing the ultimate tensile strength compared to highly crystalline materials, facilitate greater strain accommodation, preventing catastrophic brittle failure^29^. Therefore, Abelmoschus esculentus fibers exhibit promising tensile behavior characterized by a tensile strength of 13.79 MPa and less elongation before fracture. These mechanical characteristics, combined with the fiber’s natural abundance, biodegradability, and surface functionality, position them as excellent candidates for developing sustainable, bio-based materials tailored for diverse industrial and biomedical applications.

### SEM microstructure of *Abelmoschus esculentus* fiber

**Fig. 5:**
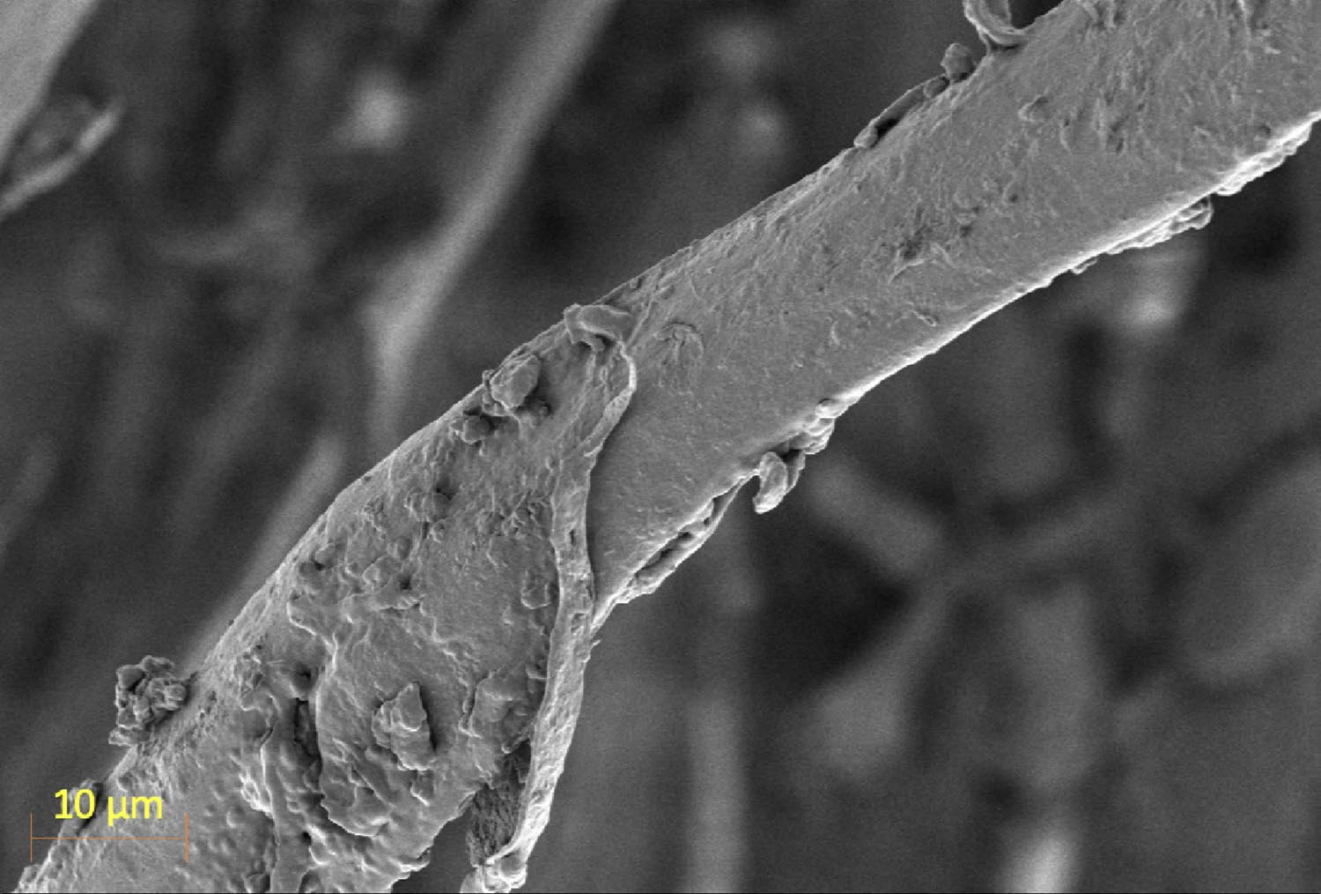
SEM image of *Abelmoschus esculentus* fibers.

The surface morphology of *Abelmoschus esculentus* fibers was thoroughly examined using scanning electron microscopy (SEM) at a magnification of 1000× under an accelerating voltage of 2.00 kV. The SEM micrograph reveals several characteristic features of natural lignocellulosic fibers, providing critical insights into their microstructural properties. The fiber surface appears relatively rough and uneven, displaying a non-uniform texture with patches of adhered layers and irregular protrusions. These surface features suggest the partial retention of non-cellulosic materials such as hemicellulose, lignin, and pectic substances even after the alkali treatment process. Closer observation shows the presence of superficial fibrillar layers partially peeling off from the main fiber body, indicating the layered nature of the fiber cell wall structure. This fibrillation of natural fibers subjected to mechanical and chemical processing, where external layers become loosened due to the breakdown of matrix materials binding the cellulose microfibrils^30^. The exposure of microfibrils and slight peeling can enhance surface area and increase fiber-matrix adhesion in composite applications, providing mechanical interlocking sites during polymer infiltration. Figure 5 shows the SEM image of *Abelmoschus esculentus* fibers.

The surface irregularities, folds, and micro-cracks observed may also result from internal stresses induced during mechanical extraction and drying processes. These microstructural features can influence the mechanical behavior of the fibers, offering points for stress concentration under load but also enhancing flexibility by allowing local deformation. Moreover, the rough surface topology improves the fiber’s wettability and provides more active sites for chemical functionalization, crucial for developing fiber-reinforced biocomposites or biomedical scaffolds. From a structural point of view, the relatively intact cylindrical shape of the fiber despite surface roughness reflects the inherent strength of the underlying cellulose microfibrillar architecture. Fiber diameter, aspect ratio, and linear density were evaluated to support the spinnability and reinforcement potential of the extracted okra fibers. SEM analysis showed fiber diameters ranging from 85 to 210 μm with an average of 142 ± 31 μm, reflecting natural fiber variability. The average fiber length was 38 ± 6 cm, resulting in aspect ratios between 180 and 420, which are suitable for effective stress transfer in composites. Linear density measurements yielded values of 18–32 tex with a mean of 24.6 ± 4.1 tex, comparable to other lignocellulosic fibers. These dimensional characteristics confirm the suitability of the okra fibers for composite and sustainable material applications. Regions rich in lignin and pectins may appear as denser, more compact zones, while cellulose-rich areas may present a more porous or fibrillated appearance^31^. This heterogeneity is advantageous in certain applications, as it allows selective chemical modifications targeting specific surface regions. Therefore, SEM analysis confirms that Abelmoschus esculentus fibers possess a microstructure characterized by surface roughness, partial fibrillation, and layered morphology, features that are favourable for enhanced mechanical interlocking and chemical reactivity in composite materials. These microstructural attributes, coupled with their natural biodegradability and mechanical flexibility, position the fibers as promising candidates for applications in eco-friendly composites, packaging materials, and biomedical devices requiring bioactive interfaces.

### Antibacterial activity of *Abelmoschus esculentus* fiber

**Fig. 6:**
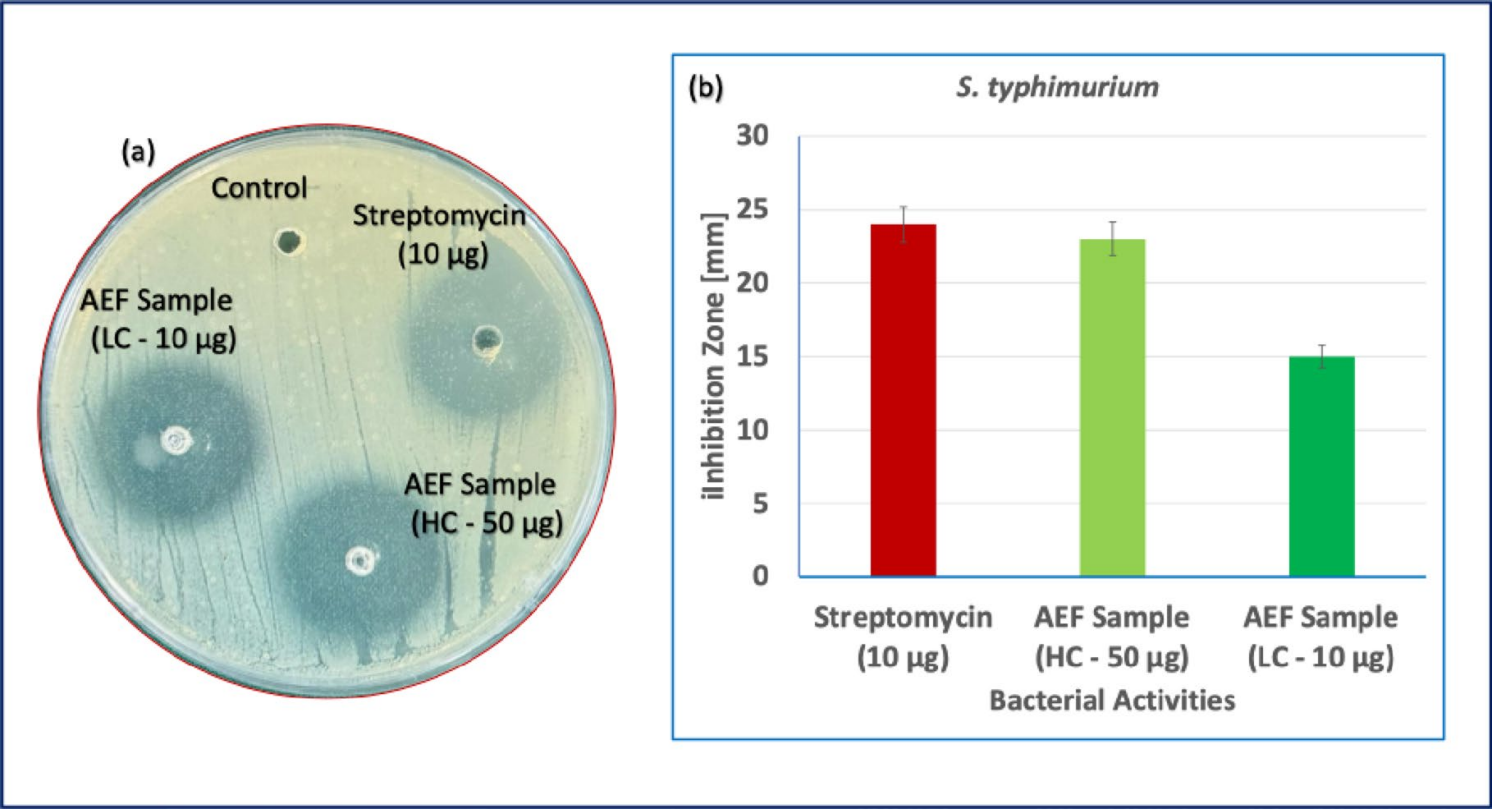
(**a**) Agar well diffusion assay showing the antibacterial activity of *Abelmoschus esculentus* fiber, (**b**) corresponding inhibition zone diameters (mm).

The antibacterial potential of *Abelmoschus esculentus* fibers (AEF) was rigorously evaluated against *Salmonella typhimurium* using the agar well diffusion method, and the efficacy was compared with that of a standard antibiotic, streptomycin (10 µg). Quantitative analysis revealed that streptomycin produced a maximum inhibition zone diameter of 24 ± 1 mm, consistent with its known broad-spectrum antibacterial activity. The AEF sample at a higher concentration (HC – 50 µg) exhibited a comparable inhibition zone of 23 ± 1 mm, while the lower concentration (LC – 10 µg) produced a smaller inhibition zone of 15 ± 1 mm. The untreated control well showed no inhibition, confirming the validity of the assay and eliminating any external contamination influence. The substantial antibacterial activity exhibited by AEF at higher concentrations can be attributed to the natural bioactive compounds inherently present in the okra fibers, such as phenolics, flavonoids, polysaccharides, and mucilage components^32^. These phytochemicals are known to disrupt bacterial cell walls, interfere with membrane integrity, and inhibit critical metabolic processes, leading to bacterial death or growth inhibition. The improved activity at the higher fiber extract concentration (50 µg) is consistent with the dose-dependent nature of bioactive compounds, where greater availability of antimicrobial constituents exerts a stronger inhibitory effect on bacterial proliferation. In contrast, the lower inhibition zone observed for the AEF sample at 10 µg concentration reflects the reduced presence of active antimicrobial molecules at this dose, resulting in partial suppression of bacterial growth but not complete inhibition. The 15 mm zone indicates that even at lower concentrations, the fibers retain intrinsic antibacterial properties, although insufficient in magnitude compared to higher dosages or standard antibiotics. Figure 6 shows (a) Agar well diffusion assay showing the antibacterial activity of *Abelmoschus esculentus* fiber, (b) corresponding inhibition zone diameters (mm).

The ability of AEF at 50 µg to approach the antibacterial efficacy of streptomycin (with only a 1 mm difference in inhibition zone) is particularly significant. Streptomycin operates by binding to bacterial R0S ribosomal subunits, causing protein synthesis inhibition. While the exact mechanism of AEF antibacterial activity requires further biochemical elucidation, it is plausible that the complex phytochemical matrix in the fibers acts via multiple pathways: disruption of bacterial membranes, generation of reactive oxygen species (ROS), and interruption of essential enzyme functions, all contributing cumulatively to bacterial inhibition. Furthermore, the physical surface features of the fibers, as observed in SEM analysis, could enhance their antibacterial action. A rough and porous surface morphology provides increased contact surface area for interaction with bacterial cells, potentially facilitating higher localized concentrations of antimicrobial agents^33^. Therefore, the study demonstrates that Abelmoschus esculentus fibers exhibit notable antibacterial activity against *Salmonella typhimurium* in a concentration-dependent manner. At higher concentrations, their efficacy is comparable to that of conventional antibiotics like streptomycin, highlighting their potential as eco-friendly, naturally derived antibacterial agents. These fibers could serve in the development of sustainable antimicrobial composites, biodegradable packaging materials, wound dressings, and medical textiles, reducing reliance on synthetic chemicals and addressing emerging concerns regarding antibiotic resistance.

### Biofilm analysis of *Abelmoschus esculentus* fibers

**Fig. 7:**
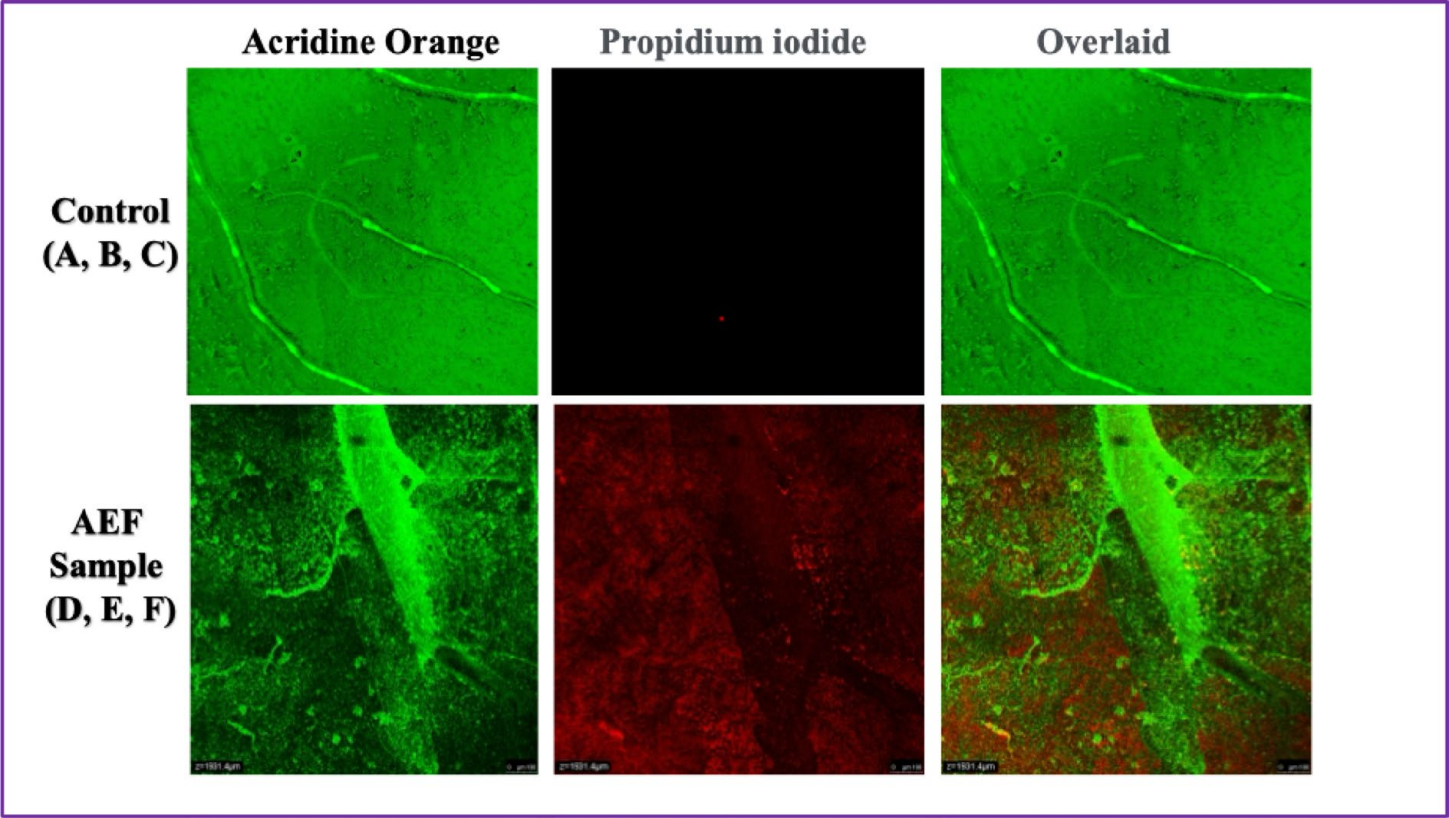
The biofilm analysis of *Abelmoschus esculentus* fibers.

The antibiofilm efficacy of *Abelmoschus esculentus* fibers (AEF), referred to here as the AEF sample, against *Salmonella typhimurium* was thoroughly investigated using confocal laser scanning microscopy (CLSM), employing acridine orange and propidium iodide dual staining for visualizing live and dead bacterial populations, respectively. Acridine orange selectively penetrates all bacterial cells, staining live cells green by intercalating with nucleic acids, while propidium iodide only penetrates cells with compromised membranes, staining them red, thereby distinguishing dead or membrane-damaged cells. The CLSM images demonstrated stark differences between untreated control biofilms and biofilms treated with the AEF sample. In the control group, biofilms exhibited dense, homogenous green fluorescence, with negligible red staining observed. This indicated that the bacterial population was predominantly viable and that a well-established, mature biofilm had formed, providing structural integrity and strong resistance to external stressors [36]. In contrast, the AEF-treated samples showed substantial red fluorescence throughout the biofilm matrix, along with a heterogeneous and disrupted distribution of green signals. The overlaid images of the treated samples revealed extensive regions of red and yellow-orange coloration, representing a mixture of dead and compromised bacterial cells. Mechanistically, the marked increase in propidium iodide uptake in the AEF-treated biofilms suggests that the fiber extracts actively compromise the bacterial cell membrane integrity, leading to loss of membrane potential, leakage of cytoplasmic contents, and subsequent bacterial cell death. This membrane disruption can be attributed to the presence of bioactive phytochemicals within the fibers, including flavonoids, polyphenols, and saponins, which are known to insert into lipid bilayers, cause destabilization, and induce oxidative stress [37]. Also, the fibrous surface morphology may facilitate mechanical interactions with bacterial cells, promoting localized membrane damage and impairing biofilm cohesion. Figure 7 shows the biofilm analysis of *Abelmoschus esculentus* fibers.

The disruption of biofilm structure observed in the treated samples can also be linked to interference with the extracellular polymeric substances (EPS) that are critical for biofilm architecture and bacterial protection. It is likely that the bioactive components of the AEF disrupt EPS synthesis or degrade existing matrix polymers, weakening the biofilm and enhancing bacterial susceptibility to environmental stress. Moreover, inhibition of quorum sensing pathways a key regulatory mechanism for biofilm development could further contribute to the observed biofilm destabilization [38]. Quantitative interpretation of the CLSM results suggests a significant reduction in viable biomass and an increase in non-viable bacterial populations following treatment with AEF. The scale bar (100 μm) emphasizes the widespread biofilm disruption across a large surface area, demonstrating the potent antibiofilm action of the fiber extracts [39]. Therefore, Abelmoschus esculentus fibers exhibit strong antibiofilm activity against *Salmonella typhimurium* by a multifactorial mechanism involving membrane disruption, inhibition of EPS stability, and potential quorum sensing interference. These findings underscore the promise of AEF fibers as natural antibiofilm agents for biomedical coatings, food packaging materials, and antimicrobial textile applications, providing a sustainable alternative to synthetic antibiofilm chemicals.

## Conclusions

In this study, fibers were successfully extracted from *Abelmoschus esculentus* plant waste stems and comprehensively characterized for their potential use in sustainable composite applications. Structural analysis using X-ray diffraction confirmed the semi-crystalline nature of the fibers, indicating a balanced presence of ordered and amorphous regions that are favorable for mechanical adaptability and chemical modification. Fourier-transform infrared spectroscopy verified the presence of cellulose, hemicellulose, and lignin functional groups, suggesting the availability of reactive sites for effective fiber–matrix interactions. Mechanical testing demonstrated moderate tensile strength and brittle behavior, supporting the suitability of the fibers for low- to medium-load biodegradable composite applications. Scanning electron microscopy revealed a rough and fibrillated surface morphology, which is advantageous for enhanced interfacial adhesion in composite systems. Also, the fibers exhibited notable antibacterial activity against *Salmonella typhimurium*, with inhibition behavior comparable to a standard antibiotic, and confocal laser scanning microscopy confirmed effective disruption of bacterial biofilm architecture. The conversion of *Abelmoschus esculentus* agricultural waste into usable natural fibers highlights an efficient recovery process achieved through retting, mechanical separation, and mild alkali treatment. Overall, the findings emphasize the dual benefits of agricultural waste valorization and the development of bio functional materials, reinforcing the potential of okra stem residues as a sustainable resource for composite, environmental, and biomedical applications.

Future work will focus on the fabrication of fiber-reinforced polymer composites to evaluate load transfer efficiency and interfacial performance under practical conditions. Long-term durability studies, including moisture absorption, aging behavior, and environmental stability, will be conducted to assess performance under service conditions. In addition, surface energy measurements, cytotoxicity evaluation, and comprehensive life-cycle assessment are proposed to further validate large-scale sustainability, environmental impact, and biomedical safety prior to industrial or clinical translation.

## Data Availability

The datasets used and/or analysed during the current study available from the corresponding author on reasonable request.
